# Potential Biomarkers of miR-371–373 Gene Cluster in Tumorigenesis

**DOI:** 10.3390/life11090984

**Published:** 2021-09-19

**Authors:** Junaid Ali Shah, Saadullah Khattak, Mohd Ahmar Rauf, Yong Cai, Jingji Jin

**Affiliations:** 1 School of Life Sciences, Jilin University, Changchun 130012, China; junaid1316@mails.jlu.edu.cn (J.A.S.); caiyong62@jlu.edu.cn (Y.C.); 2 Henan International Joint Laboratory for Nuclear Protein Regulation, School of Basic Medical Sciences, Henan University, Kaifeng 475004, China; saadullah@henu.edu.cn; 3 Department of Surgery, Miller School of Medicine, University of Miami, Miami, FL 33136, USA; mxr2481@med.miami.edu or; 4 School of Pharmacy, Changchun University of Chinese Medicine, Changchun 130117, China

**Keywords:** microRNA, miR-371–373 gene cluster, oncogene, tumor suppressor

## Abstract

microRNAs (miRNAs) are small non-coding RNA transcripts (20–24 nucleotides) that bind to their complementary sequences in the 3′-untranslated regions (3′-UTR) of targeted genes to negatively or positively regulate their expression. miRNAs affect the expression of genes in cells, thereby contributing to several important biological processes, including tumorigenesis. Identifying the miRNA cluster as a human embryonic stem cell (hESC)-specific miRNAs initially led to the identification of miR-371, miR-372, miR-373, and miR-373*, which can ultimately be translated into mature miRNAs. Recent evidence suggests that miR-371–373 genes are abnormally expressed in various cancers and act either as oncogenes or tumor suppressors, indicating they may be suitable as molecular biomarkers for cancer diagnosis and prevention. In this article, we summarize recent studies linking miR-371–373 functions to tumorigenesis and speculate on the potential applications of miR-371–373 as biomarkers for cancer diagnosis and treatment.

## 1. Background

miRNAs are the type of short non-coding RNA composed of 20 to 24 nucleotides. Wightman and Lee [1,2] first discovered two small lin-4 transcripts about 22 and 61 nt in *C. elegans* that have complementary sequences in the 3′-UTR of lin-14 messenger RNA (mRNA), providing the evidence that lin-4 transcript is responsible for the negative regulation of lin-14 mRNA, which is now known as miRNAs. MiRNAs are capable of binding to target mRNA through their 3′-UTR, rendering the target mRNA degraded or inhibiting translation of proteins [3,4], thus playing a crucial role in transcription. Recent studies have brought to light how miRNAs function in multiple regulatory functions, such as gene transcription, cell cycle control, and metabolism [3,4,5]. However, miRNAs play an important role in several cancer-relevant processes, including cell differentiation, proliferation, and migration [6]. In this review, we discuss miRNA371 (miR-371) to miR-373 and summarize current knowledge of the functions of miR-371 to 373 in intracellular biological processes. We speculate on possible roles that may contribute to tumorigenesis.

## 2. miRNAs Biogenesis

**Figure 1:**
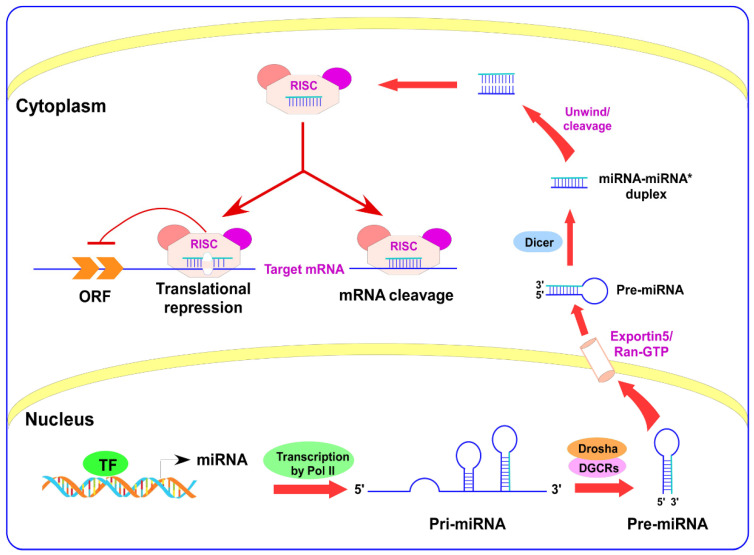
Schematic diagram of microRNA formation. ORF: open reading frame, TF: transcription factor.

miRNA biogenesis is a complex process involving multiple steps. Figure 1 shows that the RNA polymerase II enzyme initiated the transcription of miRNAs to produce primary miRNAs (pri-miRNAs) within the nucleus. [7,8,9]. Next, a complex composed of RNase III enzyme, Drosha, and the protein DiGeorge syndrome critical region 8 (DGCR8) excises the hairpin structure of pri-miRNA and turns into precursor miRNAs (pre-miRNAs), a product of about 70 nucleotides with a 2-nucleotide overhang at the 3′ end [10,11,12,13]. There is a well-established relationship between the Drosha enzyme and the Drosha protein at the hairpin base, while DGCR8 proteins receive a signal and bind to the stem of pri-miRNA for cleavage [14,15]. Consequently, pre-miRNA is transported by Exportin 5/Ran-GTP to the cytoplasm after the recognition of the overhang transcripts [16]. In the cytoplasm, pre-miRNA is recognized by the Dicer RNase III enzyme [17,18,19] that cuts the pre-miRNAs at a species-specific length [20], therefore a mature-miRNA duplex features 2-nucleotide 3′ overhang at its origin [15,21]. The latest miRNA duplex has 22 nucleotides loaded into the RNA-induced silencing complex (RISC) that directs it to its target mRNA. The targeted mRNA is degraded whenever the 3’-UTR of the mRNA and the miRNA have a stable bond. The translational repression of the targeted region can occur without any degradation of the mRNA when the complementarity of the targeted region is incomplete [22,23].

## 3. miR-371–373 Gene Cluster

**Figure 2:**
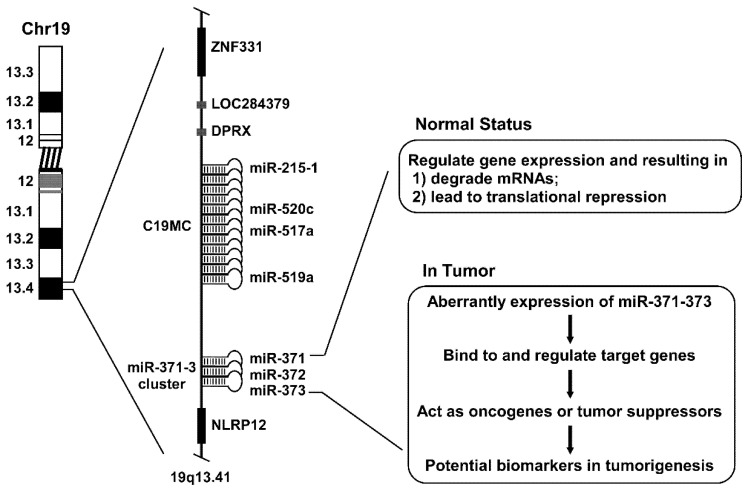
Schematic diagram of miR-371–373 gene cluster. Chr19, chromosome 19; C19MC, chromosome 19 microRNA cluster.

The miR-371–373 gene cluster is located in chromosome 19q13.4. Many oncogenic events related to HNSCC (head and neck squamous cell carcinoma) are known to reside in this region [24]. As shown in Figure 2, the miR-371–373 gene cluster can be transcribed and processed into pre-miR-371, pre-miR-372, and pre-miR-373, and four mature mRNAs are eventually formed including miR-371, miR-372, miR-373, and miR-373* [25,26]. MiR-372 and miR-373 are originally identified as the hESC (human embryonic stem cell)-specific miRNAs [26]. More specifically, miR-372 was also identified as a direct and functional target for ATAD2 (ATPase family AAA domain-containing protein 2) in hepatic carcinogenesis [27]. It has been demonstrated that miR-373 is a member of the miR-520/373 family, which consists of three miRNA clusters possessing identical seed sequences: miR-302/367, miR-371–373, and miR-520 [28,29,30]. The miR-372 and miR-373 seed sequences are different in each species. For example, the miR-290–295 cluster in mice is homologous to the miR-371–373 cluster in humans [31]. It is noteworthy that the individual pre-miRNA hairpin sequences are homologous. It can therefore be speculated that those miRNAs in cells have similar functions based on their homology and the conservation of their putative promoter elements [32].

## 4. Functions of miR-371–373 in Tumorigenesis

### 4.1. Expression Status of miR-371–373 in Different Cancer Cells

Studies confirm that the unbalanced expression of miRNAs can lead to abnormal expression of target proteins, which results in alterations to various biological processes in cells. In line with this view, aberrant expression of miRNAs has been detected in many cancers, including gastric [33], lung [34], and prostate cancer (PCa) [35], suggesting that miRNAs such as the miR371–373 gene cluster play an important role in carcinogenesis. Researchers have found that miR-371–373 expression is reduced in PCA DU145 cells [36] and pancreatic adenocarcinoma (HPAC) [37], while the expression level of miR-371–373 is increased in gastric adenocarcinoma [38] and esophageal cancer cells [39]. miR-371–373 expression is altered in tumor cells and directly affects tumor growth. Up-regulated miR-372 and miR-373, for example, play a critical role in esophageal cancer progression [40]. In addition, in HPAC, miR-372 is not only expressed at a low level but its expression is inversely correlated with ULK1 (a tumor protein UNC51-like kinase 1). Thus, overexpression of ULK1 can reverse the effects of overexpressed miR-372, suggesting the function of a miR-372/ULK1 axis in suppressing HPAC cell proliferation, migration, and invasion [41]. Moreover, the expression status of miR-373-3p is related to the overall survival rate of patients with PCa, and patients with longer survival rates have a higher expression level of miR-373-3p. Therefore, miR-373-3p is considered the best option to be used as an early diagnostic marker to differentiate PCa from benign prostatic hyperplasia [42]. A single miRNA often targets many potential proteins, thereby participating in the transcriptional regulation and genomic instability with different routes [43]. To date, accumulating evidence revealed that miRNAs can serve both as tumor suppressors or oncogenes in human cancers [44,45,46,47,48,49], creating a complication to know the exact mechanisms which lead to tumorigenesis. Taken together, the expression status of miR-371–373 gene cluster in cell and molecular pathways may be closely related to the occurrence of cancer [50].

### 4.2. Role of miR-371–373 on Cancer Stem Cells

Over the past decades, the concept of stem cells has been extended from the ESCs and adult stem cells to cancer stem cells (CSCs) [51]. CSCs belong to the subfamily of cancerous cells with capabilities of self-renewal, differentiation, and tumorigenesis, and are found in the different types of tumors [52,53]. With the improvement of experimental technology, CSCs with self-renewal ability and pluripotent differentiation potential are now possible to derive from different types of tumors in an undifferentiated state under defined culture conditions [54,55,56]. Accumulating evidence suggests that CSCs possess the unique ability to initiate and perpetuate tumor growth; this characteristic is also called “stemness” [57]. Importantly, most miRNAs are limited to specific stages in embryonic development [58,59] and play a role in embryogenesis [60]. As mentioned before, miR-371–373 gene cluster was originally identified as the hESC-specific miRNAs. According to the literature, human ESCs possess miR-371–373 gene clusters in an abundant form, suggesting that miR-371–373 genes are involved in the stemness maintenance of ESCs [61]. In line with this, miR-371–373 gene cluster in signaling pathways like the Wnt/B-catenin pathway can enhance stem cell self-renewal and their oncogenesis of various tissues [62,63,64,65]. For example, miR-372 and miR-373 enhance the stemness of colorectal cancer cells by repressing the expression of differentiation genes, such as NFkB, MAPK (mitogen-activated protein kinase-like protein)/Erk, and VDR (vitamin D receptor) [66]. Moreover, mir-372 regulates human ESC division by regulating the gap-phase checkpoints [67]. It is worth noting that miR-372 is highly expressed in human ESCs and human iPSCs (induced pluripotent stem cells)-derived primordial germ cell-like cells (PGCLCs), and human ESC cell cycle miRNA miR-372 and let-7 act antagonistically in germline differentiation from human ESCs and iPSCs. For example, knockdown of the individual miR-372 targets SMARCC1 (knockdown of the let-7 targets CMYC and NMYC suppressed PGCLC differentiation), MECP2 (methyl-CpG binding protein 2), CDKN1A (p21^Cip1/Waf1^), RBL2 (RB transcriptional corepressor like 2), RHOC (Ras homolog gene family, member C), and TGFBR2 (TGFb receptor 2) increased PGCLC production; conversely, knockdown of the let-7 targets CMYC and NMYC (two members of the oncogene Myc family) suppressed PGCLC differentiation. [68].

### 4.3. miR-371–373 Serves as Oncogenes in Human Cancers

Based on the fact that miRNAs are often deregulated in tumorigenesis, the involvement of miRNAs in regulating genes related to tumor development can be speculated. A series of recent studies have indicated that miR-371–373 gene cluster plays an important role in various types of cancers by targeting certain genes [69,70] (Table 1). Importantly, from the perspective of the metastatic function of cancer cells, miR-371–373 clusters can act as both tumor suppressors and oncogenes by monitoring migration and invasion [71]. 

LATS2 (large tumor suppressor kinase 2) gene, a homolog of the LATS tumor suppressor family, plays a critical role in controlling cell cycles and tumor development through hippo pathway, p53, and Ras-ERK signal transduction [72]. Therefore, it is not difficult to understand that the aberrant expression of LATS2 in cells may lead to tumor occurrence. In line with this, in gastric adenocarcinoma cells, highly expressed miR-372 produced the proliferation and migration of cancer cells by suppressing the LATS2 [38]. Moreover, the down-regulation of LATS2 by miR-372 prolonged the survival of esophageal squamous cell carcinoma [73], suggesting that miR-372 and miR-373 may act as oncogenes in cancer cells. Subsequent research confirmed this view. In testicular germ cell tumors (TGCTs), the miR-371–373 cluster is involved in overruling cellular senescence induced by oncogenic stress, therefore allowing cells to become more malignant [25,73]. The high-throughput microRNAome analysis further confirmed that the miR-371–373 cluster is implicated in regulating the differentiation of stem cells and retained in TGCTs [25]. More in-depth research suggests that LATS2 and P53 are pivotal genes in the tumorigenesis of esophageal and many other cancer cells are targeted by miR-371–373 [74]. For instance, miR-372 and miR-373 neutralize p53-mediated CDK (cyclin) inhibition by suppressing the *LATS2* in TGCTs, indicating that miR-372 and miR-373 may act as oncogenes participating in the development of human TGCTs by numbing the p53 pathway [73]. Later research also found that miR-373 in vivo oncogenic function was mediated by its negative regulation of TP53INP1, LATS2, and CD44 [75]. On the other hand, zinc importer ZIP4 transcriptionally induces miR-373 in PCa through activating the zinc-dependent transcription factor CREB, suggesting that the ZIP4-CREB-miR-373 signaling axis promoting pancreatic cancer growth [76].

In addition to LATS2, multiple genes have been confirmed to be target genes of the miR-371–373 cluster and act as oncogenes in tumorigenesis. In human gastric carcinoma cells, miR-372 down-regulates TNFAIP1 (tumor necrosis factor, α-induced protein 1) and further activating the NFkB signaling pathway, therefore increasing cell proliferation, indicating the oncogenic function of miR-372 [77]. It has been found that intracellular FGF9 (fibroblast growth factor 9), a member of the FGF family, is abnormally expressed in various cancers including lung, prostate, ovarian, and endometrioid cancers [78,79,80]. FGF9 is a downstream target of Wnt signaling in ovarian endometrioid adenocarcinomas with oncogenic properties [81]. Based on these findings, miR-372-3p promotes cell growth and metastasis by targeting FGF9, suggesting that the miR-372-3p acts as an oncogene in lung squamous cell carcinomas (LSCCs) [82]. Moreover, miRNAs 373 and 520c are downregulated in PC and enhance the invasion of PC cells in vitro via suppressing CD44 translation [83]. miR-373 suppresses tumor invasion and metastasis in epithelial ovarian cancer (EOC) by targeting Rab22A oncogene [84].

### 4.4. miR-371–373 Serves as Tumor Suppressors in Human Cancers

Accumulating evidence reveals that the miR-371–373 cluster can serve both as a tumor suppressor and an oncogene in human cancers. What needs to be emphasized is that the complementary sequence of miR-372 and miR-373 was found in multiple genes. For example, a putative miR-373 target site in the promoter of E-cadherin and *CSDC2* (cold shock domain-containing protein C2) was identified [93]. As a result, miR-373 increases the expression levels of *E-cadherin*, thereby inhibiting migration of lung non-small-cell cancer (LNSC) A549 cells [50]. According to the data from Ding and colleagues, the miR-520/372/373 families can target the 3′-UTR of SPOP (speckle-type POZ protein) which up-regulated in over 90% of renal cell carcinoma (RCC) and suppress the SPOP protein expression, thus leading to elevation of PTEN and DUSP7 levels and suppressing the progression of RCC in vitro and in vivo [94]. In estrogen receptor-negative breast cancer, the miR-520/373 family is a strong inhibitor of the NFkB and TGFB signaling pathway through direct targeting p65 [30]. In another case, up-regulation of miR-373 in pancreatic cancer cells can repress the transforming growth factor B (TGFB)-induced EMT, thus playing an important role to inhibit the invasiveness of cancer cells [37]. Furthermore, both aldehyde dehydrogenase A1 (ALDH1A1) and TGFBR2 are identified as potential target genes of the miR-371–373 cluster. Stably overexpressing the entire miR-371–373 cluster successfully reduced the tumor initiation and metastatic growth capacity in different colon TIC cultures by repressing TGFBR2 [95]. In PCa, lower expressed miRNA-373-3p leads to the progression of cancer cells by affecting AKT1 (AKT serine/threonine kinase 1) [42]. Furthermore, miR-373 directly targets and inhibits EGFR expression, resulting in a low expression level of VE-cadherin (vascular endothelial-cadherin), thereby suppressing the activity of MMPs by inhibiting the PI3K/AKT pathway [96].

IGF2BP1 (insulin-like growth factor 2 mRNA-binding protein 1), a member of the RNA-binding proteins, was identified as a target of several miRNAs including miR-372, miR-494, and miR-625. Experimental results demonstrate that a high level of intracellular miR-372 can bind with its target site at the 3′-UTR region of IGF2BP1, thereby causing the repression of IGF2BP1 expression [97]. Silencing IGF2BP1 reduces cell proliferation and promotes apoptosis in hepatocarcinoma cells [86], suggesting that miR-372 may act as a tumor suppressor by inhibiting IGF2BP1. Consistent with this, overexpression of miR-372 in cancer cells blocks autophagy activation and inhibits in vivo tumor growth through regulating sequestosome1 (SQSTM1 or p62) [91]. Levels of miR-372 and p62 are inversely correlated in human HNSCC tissues. More specifically, miR-372 can suppress p62, thus increasing ROS (reactive oxygen species) and motility in HNSCC cells by inhibiting phase II detoxification enzyme NADPH Quinone oxidoreductase 1 (NQO1) [98]. In addition, up-regulated miR-372 in glioma cell lines and tissues can be suppressed by directly targeting PHLPP2 (PH domain and leucine-rich repeat protein phosphatase 2), and leads to the inhibition of cell proliferation and invasion, this further induces G1/S arrest, apoptosis, and prevention of the PI3K/Akt pathway. Moreover, an in vivo study of xenograft mouse clearly discovered the suppressive effects of miR-372 knockdown on tumor growth [99]. Furthermore, FXYD6 (FXYD domain-containing ion transport regulator 6) is targeted by miR-372. It has been known that FXYD6 is frequently up-regulated in cancer cells such as cholangiocarcinoma and osteosarcoma [100], and this higher level of FXYD6 can be targeted by miR-372-3p in osteosarcoma, thereby inhibiting the growth and metastasis of cancer cells [68]. Because of this, FXYD6 has become a potential biomarker in osteosarcoma.

### 4.5. miR-371–373 Targets Cell Cycle-Related Genes

At present, it is generally believed that the continuous division of cells being receiving oncogenic signals is the most common route to cause tumorigenicity [101]. Thus, alteration in the cell cycle machinery performs a critical role in tumor generation, especially some cell cycle regulatory proteins that play an essential role in tumor development. The growing research reported that miRNAs are deeply connected with proteins involved in cell cycle regulation [88]. Gene expression studies in nasopharyngeal carcinoma (NPC) TW01 cells found that CDKN1A/p21, INCA1 (Cyclin A1 interacting protein 1), LATS2, and BIRC (baculoviral inhibitors of apoptosis repeat-containing) are up-regulated, and CDK2 (cyclin-dependent kinase 2), Cyclin A1, TP53, BAX (bcl-2-associated X protein), and BCL2 (B-cell CLL/lymphoma 2) are down-regulated by miR-372. Further experimental research confirmed that miR-372 causes cell cycle arrest at the S phase, and may also act as a tumor suppressor in cell cycle progression of TW01 cells via the down-regulation of CDK2 and CCNA1 as well as the up-regulation of CDKN1A/p21 and INCA1 [89]. In line with this, the experimental data so far strongly suggest that overexpressed miR-371–3 cluster influences the most important genes of the cell cycle, such as CDK2, CDK4, and CDK6, thereby leading to malignant progression through interrupting cell cycle and differentiation of cells [87]. For example, overexpression of miR-371–373 accelerates the progression of cell division and inhibition of apoptosis in malignancies in numerous cancers such as colon cancer stem cells, lung cancer, breast cancer, liver cancer, and germ cells [87,102,103]. In human cervical cancer HeLa cells, CDK2 and Cyclin A1 are known to be the direct targets of miR-372 and negatively regulate proliferation and cell cycle progression [87]. Similar results were obtained from the experiments in endometrial adenocarcinoma (EC). miR-372 suppresses cell proliferation, migration, and invasion, and leads to a G1 phase arrest by down-regulating Cyclin A1 and CDK2. Bioinformatic predictions further found that RhoC was a possible target of miR-372, suggesting that miR-372 suppresses tumorigenesis and development in EC [104]. In contrast, reduction of the miR-372 level in hESCs causes attenuation of cell division. In more detail, miR-372 can reduce CDKN1A/p21 levels in hESCs, preventing it from reaching the level of effective G1 checkpoint components that can regulate the activity of the CycE/CDK complex [66].

### 4.6. miR-371–373 Gene Cluster as an Epigenetic Regulator in Cancer

The definition of the word “epigenetics” has evolved continually as science has progressed. Epigenetics is now described as the “inheritance of mitosis and/or meiosis” without alterations to the DNA sequence [105,106,107]. There are many ways of epigenetic regulation, such as DNA methylation, histone modification, and chromatin remodeling. However, no matter what kind of regulation ultimately leads to changes in chromatin structure, thereby affecting the transcriptional regulation of genes [108,109]. Recent accumulating evidence assumes that non-coding RNAs like miRNAs also have a functional role in different molecular mechanisms that assist epigenetics [110]. Research data have demonstrated that miRNAs are involved in the initiation of several processes of carcinogenesis by impacting the transcriptional regulation of genes that associate with epigenetic machinery [111,112,113]. 

DNA methylation is the main epigenetic feature of DNA and plays a key role in the regulation of gene transcription and maintaining genome stability. Changes in DNA methylation can disrupt transcription levels and lead to pathological phenomena including cancer [114,115]. For example, JMJD2A, a demethylase for histone H3K9/K36, is frequently overexpressed in several tumors such as liver cancer. A higher level of JMJD2A inhibits the DNA-damage repair by reducing homologous recombination (HR) repair. Simultaneously, JMJD2A can be negatively regulated by SIRT2 in cancer [116,117]. Interestingly, JMJD2A inhibits the methylation of the miR-372 promoter region and promotes the recruitment of p300 and RNApolII on it. In contrast, miR-372 influences the editing of JMJD2A and prompts a novel transcript (JMJD2AΔ) of JMJD2A. Noting that JMJD2A inhibits CDKN1A/p21 in cell cycle progress through JMJD2AΔ dependent on miR-372, this further facilitates cell cycle progress via the Pim 1-pRB-CDK2-CyclinE-c-Myc pathway [118], suggesting the oncogenic function of JMJD2A. Moreover, the epigenetic regulation of miR-373 in hilar cholangiocarcinoma was found by bioinformatic prediction. MBP (methyl CpG binding protein) mediated high-methylation of promoter-related CpG islands are consistent with miR-373 inhibition [119]. Similarly, miR-373 negatively regulates MBD2 (methyl-CpG-binding-domain protein 2) expression [120]. In line with this, depletion of methyltransferases DNMT3B and DNMT1 in colorectal cancer HCT116 cells caused transcriptional inactivation of miR-373 through decreasing CpG islands methylation [121]. 

Transcriptional silencing of miRNAs in cancer is one of the mechanisms of epigenetic changes. In addition to the miR-372 and miR-373 being silenced by DNA hypermethylation, its expression may also be regulated by histone modifications. For example, down-regulated miR-373 in LNSC A549 and Calu-6 cells can be restored by histone deacetylase (HDAC) inhibitors SAHA (suberoylanilide hydroxamic acid) and trichostatin A (TSA), and this further results in the reduction of miR-373 target gene IRAK2 and LAMP1, thereby attenuating cell proliferation and invasion [90]. In another case, miR-520 and miR-373 up-regulate MMP9 (matrix metalloprotein 9) by directly targeting the 3′-UTR of mTOR and SIRT1 mRNAs in human fibrosarcoma HT1080 cells, and further activating the Ras/Raf/MEK/Erk signaling pathway and NF-κB, thereby enhancing cell growth [29]. 

### 4.7. miR-371–373 as the Target of New Chemotherapeutic Drugs

Epigenetic alterations are often reversible, which has led to the emergence of the promising field of epigenetic therapy. Research data so far have shown that most miRNAs, as versatile non-coding RNAs, play a vital role in gene expression, thereby regulating the different physiological and cancer-related processes in cells [92,122]. Compared with antibody-based targeting of specific proteins, miRNAs—a class of endogenous small non-coding single-stranded RNAs—have higher specificity, sensitivity, and price advantage [123]. Therefore, although the DNMTs, HDACs, and HATs have been used as biomarkers in research and clinical applications, miRNAs with better accuracy involved in the regulation of post-transcriptional gene expression are more attractive as biomarkers [124]. 

In 1994, Murray and his collaborators reported that malignant germ cells tumors (GCTs) overexpress miRNAs of the m371–373 and m302 clusters, regardless of the patient’s age, histologic subtype, or location [125]. Interestingly, the elevated cancer-related miR-371–373 serum levels in GCT patients cleared quickly after treatment, suggesting that miR-371–373 offer greater sensitivity and specificity for diagnosing and monitoring malignant GCTs [126,127]. In GCTs, β-HCG (β subunit of human chorionic gonadotropin), AFP (alpha-fetoprotein), and LDH (lactate dehydrogenase) are clinically used as serum markers to diagnose tumors but have limitations in sensitivity and specificity, especially in certain tumor subtypes, such as SE and EC [128]. Therefore, miRNAs have become more accurate and reliable biomarkers for tumor diagnosis and prognosis. Dieckmann and colleagues tried to use serum miR-371a-3p expression levels as biomarkers to accurately assess TGCT, and to replace the existing conventional biomarkers such as AFP, β-HCB, and LDH [129]. Afterward, a large number of published data explored the novelty of miRNA clusters. However, so far, it is considered that miR-371a-3p are the most suitable for the overall performance of serum samples with 88.7% sensitivity and 93.4% specificity, and the expression level is also reduced after chemotherapy, so it is being used for diagnosis in liquid biopsies of cancer patients, for both diagnostic, prognostic, and predictive purposes such as germ cell neoplasia in situ (GCNIS) patients [130,131]. 

Harel and colleagues report that the expression level of miR-512 and miR-373 secreted in exosomes are increased by treating lung cancer cells with inhibitors of DNMT and HDAC (5′-aza-deoxycytidine and TSA). Interestingly, this re-expression of both miR-512 and miR-373 sensitizes lung cancer cells to cisplatin and restricts tumor growth. Subsequent experimental data confirmed that miR-373 directly targets and represses *RelA (p65)* and *PIK3A*, thus facilitating cell death upon treatment with cisplatin [85]. 

## 5. Conclusions and Perspectives

Different types of cancer have been associated with the abnormal expression of the miR-371–373 gene cluster. By regulating the expression of the targeted genes, miR-371–373 acts as both an oncogene and a tumor suppressor. miR-371 –373 is closely associated with tumorigenesis, especially at its positioning on chromosome 19q13.4, where many oncogenic events associated with HNSCC reside. During tumorigenesis, aberrantly expressed miRNAs in serum can be detected, providing a valuable way to track cancer progression. miR-371–373, a gene highly expressed in GCTs, such as TGCT, is rapidly reduced after chemotherapy, suggesting that it might be the most appropriate biomarker for detecting patient response to chemotherapy. To more effectively and safely understand the principles of miRNA-based cancer therapy, more in-depth investigation is necessary.

## Figures and Tables

**Table 1:** miR-372 and miR -373 regulate target genes involved in tumorigenesis.

miRNAs	Tumor Tissues or Cancer Cells	Target Genes	Exp. of Targets	Consequent	Ref
mir-372	Prostate cancer	p65	Down	Acts as a tumor suppressor	[30,85]
	Liver carcinoma	ATAD2	Down	Acts as a tumor suppressor	[32]
	Gastric carcinoma, ESCC	LATS2	Down	Acts as an oncogene	[38,72]
	Colorectal cancer	NFkB, MAPK/Erk, and VDR	Down	Enhances the stemness	[65]
	Osteosarcoma tissues	FXYD6	Down	Acts as a tumor suppressor	[68]
	TGCT	LATS2	Down	Acts as an oncogene	[73,74,75]
	Gastric cancer	TNFAIP1	Down	Acts as an oncogene	[77]
	Lung carcinoma	FGF9	Up	Acts as an oncogene	[82]
	SCC	p62	Up	Acts as a tumor suppressor	[86]
	Glioma tissues	PHLPP2	Up	Acts as an oncogene	[87]
	Cervical, liver cancers, EC, HeLa, germ cell,	CDK2,Cyclin A1	Down	Acts as a tumor suppressor	[88,89]
	Renal cell carcinoma	MBD2	Down	Acts as a tumor suppressor	[90]
mir-373	Fibrosarcoma	mTOR, SIRT1	Down	Acts as an oncogene	[29]
	Breast cancer	TGFB, NFkB	Up	Acts as a tumor suppressor	[30]
	Esophageal cancer	LATS2	Up	Acts as an oncogene	[71]
	Testicular germ cell tumors	LATS2	Down	Acts as an oncogene	[73,74,75]
	Pancreatic cancer	ZIP4	Up	Acts as an oncogene	[76]
	Prostate cancer	CSDC2	Up	Acts as a tumor suppressor	[83]
	Prostate cancer tissue	CD44	Down	Acts as an oncogene	[83]
	Ovarian cancer	Rab22A	Down	Acts as a tumor suppressor	[84]
	Prostate cancerA549 cell	E-cadherin	Up	Acts as a tumor suppressor	[83,84]
	Urinary bladder cancer	EGFR	Up	Acts as a tumor suppressor	[91]
	Hilar cholangiocarcinoma	MBD2	Down	Acts as a tumor suppressor	[90]
	Lung cancer	IRAK2, LAMP1	Down	Acts as a tumor suppressor	[90,92]
	Lung cancer	RelA, PIK3CA	Down	Acts as a tumor suppressor	[85]

ESCC, esophageal squamous cell carcinoma; SCC, squamous cell carcinoma; TGCT, testicular germ cell tumors.

## Data Availability

Not applicable.

## Abbreviations

miR-371–373	microRNA-371–373genecluster
3′-UTR	3′-untranslatedregion
ESCs	embryonicstemcells
DGCR8	DiGeorgesyndromecriticalregion8
pri-miRNAs	primarymiRNAs
pre-miRNAs	precursormiRNAs
RISC	RNA-inducedsilencingcomplex
HNSCC	headandnecksquamouscellcarcinoma
ATAD2	ATPasefamilyAAAdomain-containingprotein2
PCa	prostatecancers
HPAC	pancreaticadenocarcinoma
ULK1	atumorproteinUNC51-likekinase1
CSCs	cancerstemcells
PGCLCs	primordialgermcell-likecells
TGFBR2	TGFbreceptor2
LATS2	largetumorsuppressorkinase2
TGCTs	testiculargermcelltumors
CDK	cyclin
FGF9	fibroblastgrowthfactor9
LSCC	lungsquamouscellcarcinoma
EOC	epithelialovariancancer
LNSC	lungnon-small-cellcancer
RCC	renalcellcarcinoma
AKT1	AKTserine/threoninekinase1
IGF2BP1	insulin-likegrowthfactor2mRNA-bindingprotein1
FXYD6	FXYDdomain-containingiontransportregulator6
HDAC	histonedeacetylase
TSA	trichostatinA
GCTs	germcelltumors
ORF	openreadingframe
TF	transcriptionfactor.
